# Privacy-Preserving Collaborative Population Stratification with Dynamic Algorithm and Hyperparameter Selection

**DOI:** 10.1145/3765612.3767209

**Published:** 2025-12-10

**Authors:** Maryam Ghasemian, Lynette Hammond Gerido, Erman Ayday

**Affiliations:** Case Western Reserve University, Cleveland, Ohio, USA; Case Western Reserve University, Cleveland, Ohio, USA; Case Western Reserve University, Cleveland, Ohio, USA

**Keywords:** Population Stratification, Clustering, Principal Component Analysis, Privacy, Differential Privacy, Membership Inference Attack, Data Mining, Machine Learning

## Abstract

We present a privacy-preserving *selection layer* for collaborative population stratification under ϵ-local differential privacy (LDP). Rather than fixing a single pipeline (e.g., PCA+K-Means with preset K), our framework lets parties choose among three DP pipelines: *PCA→Noise*, *Noise→PCA*, and *Noise*-*Only*, according to their resources, and has an honest-but-curious server aggregate only DP shares to *automatically* select the clustering algorithm (K-Means, GMM, or Hierarchical) and K that maximize internal metrics (Silhouette, Calinski–Harabasz, Davies–Bouldin). Because selection operates on DP data, it adds no further privacy loss. On *openSNP* (942 samples, 28,396 SNPs), the PCA-augmented pipelines yield higher utility and substantially lower communication and runtime than *Noise-Only*, and the recommended configuration consistently outperforms fixed baselines. Membership-inference attack power remains markedly lower for PCA-based pipelines across privacy budgets ϵ. In this paper, experiments are limited to two collaborating parties; extensions to multi-site collaboration are left for future work.

## 1 Introduction

Population stratification, the presence of subpopulation structure within genetic data, can confound downstream analyses such as association studies and clustering-based phenotype prediction. In collaborative settings, multiple parties (researchers) each hold high-dimensional genotype or phenotype datasets and wish to jointly stratify the combined population without revealing sensitive individual-level information. Recent privacy-preserving approaches show that secure multi-party computation (MPC) or local differential privacy (LDP) can make collaborative clustering feasible. Yet nearly all of these protocols hard-code a single algorithm and set of hyper-parameters–most commonly principal-component analysis (PCA) followed by K-Means with a preset K (number of clusters). Because clustering performance is highly data dependent, the “one-size-fits-all” choice often leads to sub-optimal subgroup discovery. A framework that learns which algorithm and parameters to use after seeing the noisy data would close this configuration gap.

In this work, we present a framework for determining the optimal clustering parameters for population stratification in high-dimensional genomic data under an *honest-but-curious* server model. Also, to accommodate the diverse resources and preferences of participating parties (researchers), we introduce three ϵ-DP pipelines, each offering a distinct trade-off between researchers and server computational budgets, utility and privacy:

1. **PCA→Noise**: each party transforms its data onto r≪d local principal components, then adds Laplace noise to the projected coordinates before sharing. Here, the local computation is small which reduces the communication cost.
2. **Noise→PCA:** each party first adds Laplace noise to raw data, then applies PCA on the noisy data before sharing. The initial perturbation satisfies ϵ-DP immediately, while the follow-up projection trims data-share size without additional privacy loss.
3. **Noise-Only:** each party adds Laplace noise to full d-dimensional raw data and shares the full noisy dataset without dimensionality reduction (no PCA overhead).

Because no single pipeline dominates the others across all scenarios, we view them as complementary options that collaborators can select according to their hardware limits, accuracy targets and privacy budgets.

After collecting the ϵ-DP shares, an honest-but-curious server searches a menu of clustering algorithms— *K-Means, Gaussian Mixture Models*, and *Agglomerative Clustering*—over a range of cluster counts. Each configuration is scored with a weighted composite of the silhouette, Calinski–Harabasz and Davies–Bouldin indices; the configuration with highest score is returned to all parties. Crucially, the entire search runs on noisy data, so no additional privacy loss is incurred.

Through experiments on real genomic data, we show that (1) Algorithm selection works. The server’s top-ranked configuration later attains the best internal metrics under every pipeline and privacy budget (ϵ) tested. (2) Privacy guarantees hold. All three pipelines satisfy ϵ-DP with no extra loss during algorithm-parameter selection. Noise-Only pipeline noticeably has higher membership inference attack power, whereas in the PCA-augmented pipelines the membership inference attack power stays low, providing better privacy protection. (3) Resource trade-offs are clear. PCA-augmented pipelines cut server runtime rigorously and provide better privacy compared to Noise-Only.

Our main contributions are:

- A collaborative stratification framework accommodating multiple DP pipelines;
- A comparative analysis of PCA-before-noise vs. noise-before-PCA vs. Noise-Only under identical ϵ;
- A weighted scoring method for algorithm selection and parameter tuning on server-side aggregated data.

## 2 Background and Related Work

Population stratification is commonly revealed by projecting genotypes onto a low-dimensional principal-component (PC) space and then clustering the PC scores to expose substructure. In human genetics, the first few PCs capture broad ancestry axes, which is why PCA [1, 16] has become the pre-processing step for stratification [14]. Clustering on PC scores is typically performed with centroid-based methods (K-Means), model-based mixtures (GMM), or agglomerative hierarchical clustering; in practice, many pipelines default to PCA followed by K-Means with a fixed K, which can be suboptimal when the true structure deviates from that choice.

We adopt local differential privacy (LDP) [3, 4, 13] to protect each party’s contribution before any data leave the site. A randomized mechanism ℳ satisfies ϵ-LDP if, for all inputs $x,x^{\prime }$ differing in one individual and for all measurable outputs $y,\text{Pr}[ℳ(x)=y]\leq e^{\epsilon }\text{Pr}\left[ℳ\left(x^{\prime }\right)=y\right]$. We instantiate LDP with the Laplace mechanism, adding i.i.d. noise calibrated to the $\ell_{1}$-sensitivity Δ of the released statistic: for a scalar coordinate f(x), release $\hat{f}(x)=f(x)+\eta$ with η~Lap(Δ/ϵ) [5]. Post-processing invariance ensures that deterministic transforms (e.g., applying a fixed PCA projection after noising) do not consume additional privacy budget.

Private PCA has been studied in both output-perturbation and statistically optimal formulations, showing that concentrating privacy on a low-dimensional subspace preserves signal more effectively than perturbing all d raw features [2, 15]. Our setting leverages a public PCA model trained by an honest-but-curious server on non-overlapping data to ensure that all parties project into a common r-dimensional basis, making their shares comparable. In contrast to prior work that fixes a single clustering algorithm and K a priori, we evaluate multiple clustering families over candidate K and select the configuration that maximizes a composite of internal metrics (silhouette, Calinski–Harabasz, Davies–Bouldin) computed on already DP-protected shares. This yields a data-driven, privacy-preserving approach to collaborative stratification that adapts algorithm and hyperparameters to the observed structure rather than assuming them in advance.

## 3 System and Threat Models

### 3.1 System Model

In our collaborative population stratification framework, there are two key entities. An honest-but-curious server and two or more parties (researchers). Honest-but-curious server helps parties to find best algorithm/parameter combo for their collaborative population stratification. The server might publish a global PCA model—computed on a public dataset— only if parties choose to employ PCA in their computations. Parties then apply one of three local pipelines under an ϵ-DP guarantee. Finally, the server aggregates shares to perform clustering and parameter selection. Table 1 contains a list of symbols and notations used throughout this paper. Formally, the components are:

- **Parties (Researchers):** A set of n institutions, each holding a private dataset $D_{i}\subset R^{d}$ of high-dimensional genetic or phenotypic measurements. Each party applies one of the three DP scenarios to produce a DP-processed data $S_{i}$ and send that to the server. Their goal is to identify population sub-structure while keeping raw genotypes private.
- **Honest-but-curious Server:** The server aggregates transformed or noisy data from all parties, performs clustering, and returns the recommended algorithm and optimal parameters back to the parties.
- **Optional Public PCA Model:** For the two PCA-augmented pipelines (PCA→Noise and Noise→PCA) the server publishes a PCA model $\text{Q}\in R^{d\times r}$, trained on public data. The Noise-Only pipeline ignores this component.

We evaluate the ϵ-DP privacy of all three settings in sections 5 and 6.

### 3.2 Threat Model

In our setting, we assume that each research party is honest: they possess legitimate genomic or phenotypic datasets and faithfully follow the protocol. The only adversary is the server, which we model as “*honest-but-curious*”: the server correctly performs all computations–training the global PCA model on a public dataset, collecting each party’s differentially private share, running the clustering and validation routines, and returning its recommendation–but it may examine the metadata (i.e., the noisy feature or PCA-score vectors) it receives in an effort to learn more about individual participants.

Genomic datasets are vulnerable to several well-known inference attacks. In a membership-inference attack [11], an adversary who possesses an individual’s genomic profile attempts to determine whether that person’s data were included in a target dataset. In an attribute-inference attack [12], the adversary uses auxiliary information to predict sensitive traits (e.g., disease status or ancestry) of participants. In de-anonymization attacks [9], the goal is to link an identity–often available from public records–to a record in the dataset. Of these, membership inference is the most relevant in our considered setting: our *honest-but-curious* server might try to use the noisy shares $S_{i}$ to decide whether a particular individual contributed data to one of the parties.

To combat these attacks, we require that each party’s transmitted share satisfy ϵ-local differential privacy. By calibrating Laplace noise to the global sensitivity of each coordinate—whether in the raw d-dimensional space or in the r-dimensional PCA projection—we ensure that observing $S_{i}$ reveals almost no information about the presence or absence of any single record beyond what is permitted by ϵ.

Our model relies on three core assumptions. First, each party correctly implements its DP pipeline—using the server-provided PCA model when required, computing sensitivities from known feature ranges, and sampling noise from the Laplace distribution with the agreed parameters. Second, there is no collusion between any party and the server; parties never share raw data with the server. Third, feature ranges (and hence sensitivities) for both raw and PCA-transformed data can be safely bounded, enabling correct noise calibration. Given these conditions, any further clustering, metric computation, or algorithm-and-parameter selection performed by the server is on DP-protected data and incurs no additional privacy loss.

In the following, we list the specific adversary goals for each of the three settings we present:

#### PCA→Noise.

The server observes only r noisy components. Although dimensionality reduction limits exposure, an honest-but-curious server may exploit auxiliary projections or public PCA bases to reconstruct lower-variance features.

#### Noise→PCA.

Noise is first injected in the d-dimensional space; subsequently projecting onto top PCs concentrates signal-to-noise ratio along principal axes. An adversary could thus focus inference on retained dimensions where noise impact is minimal.

#### Noise-Only.

Full d-dimensional noisy data provides maximal visibility. In this setting, the server can apply reconstruction or de-noising techniques on each feature independently, increasing membership-inference risk when ϵ is large enough.

## 4 Proposed Framework

**Figure 1:**
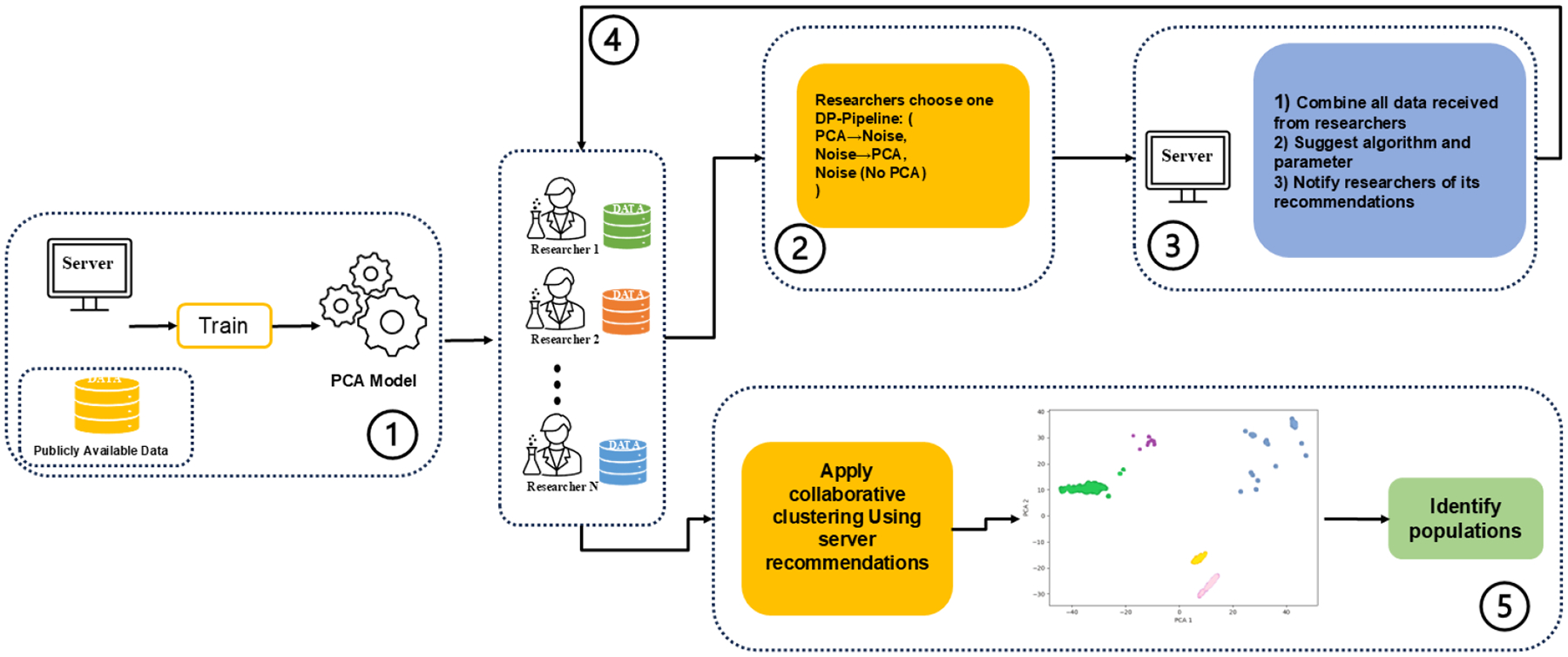
System model for privacy-preserving collaborative population stratification. (1) The server trains a global PCA model on a publicly available reference dataset and publishes the projection matrix and privacy budget ϵ to all parties. (2) Each researcher applies the shared PCA basis and one of three DP pipelines, PCA→Laplace noise, Laplace noise→PCA, or Noise-Only—to generate an ϵ-DP data share. (3) The server collects all sanitized shares, concatenates them, and fits K-Means, Gaussian Mixture Models, and Agglomerative Clustering across a range of cluster counts. It then computes and normalizes silhouette, CH, and DB indices into a composite score and returns the optimal algorithm and cluster number. (4) Researchers receive the recommendation and apply the chosen clustering configuration to their combined data. (5) The final clustering partition defines the inferred subpopulations for downstream analyses.

We implement and compare three complementary locally differentially private (LDP) pipelines for collaborative clustering. Based on the resources and (privacy utility) preferences of the parties, two pipelines combine PCA with Laplace noise in different orders, and one omits dimensionality reduction. All three pipelines satisfy an ϵ-LDP guarantee, but occupy different points on the privacy ↔ utility ↔ complexity trade-off. Figure 1 sketches the workflow.

### 4.1 The Public PCA Model

When a pipeline uses PCA, all parties must project their data into the same low-dimensional model so their vectors are comparable, otherwise, clustering would be meaningless. The server therefore trains a PCA model Q once on a public dataset and shares Q with all parties; publishing Q costs no privacy because it contains no private rows from the parties. Pipelines that skip PCA (Noise-Only) ignore this step.

### 4.2 Pipeline 1: PCA→Noise

Each party standardizes its data, projects onto the top r components of Q, and then adds independent Laplace noise to each projected coordinate with scale $\Delta_{j}/\epsilon$ (where $\Delta_{j}$ is the sensitivity of the component j). The resulting noisy PC scores are the party’s private share to the server. Adding noise after projection typically yields higher utility: in lower dimension r≪d per-coordinate $\ell_{1}$-sensitivity (and noise scale) is smaller, so signal is preserved better than perturbing all d features [2, 6]. From a clustering standpoint, low-dimensional, noisy PCA scores retain subpopulation structure. Private PCA mitigates the curse of dimensionality; utility scales with intrinsic dimension r, not d [10]. Theory also shows near-optimal error with minimal samples [15], so focusing privacy on top r preserves signal-to-noise.

### 4.3 Pipeline 2: Noise→PCA

This pipeline reverses the order of transformation and noise. Each party first adds Laplace noise to every raw SNP coordinate in ${\tilde{D}}_{i}$, thereby satisfying ϵ-DP immediately. It then applies the same public PCA model Q described above, yielding a reduced matrix $P_{i}^{\text{noisy}}\in R^{n_{i}\times r}$. As before, these noisy principal-component scores comprise the local DP share sent to the server.

### 4.4 Pipeline 3: Noise-Only (No PCA)

In the simplest pipeline, parties inject Laplace noise into every original feature of ${\tilde{D}}_{i}$ and share the full $n_{i}\times d$ noisy matrix. No dimensionality reduction is performed, and the server clusters in the raw feature space. Dropping PCA preserves all signal and can reach high utility once ϵ is generous, but it also pushes server-side runtime high and exposes a larger attack power in membership-inference tests (section 6.2).

### 4.5 Server-Side Clustering and Parameter Tuning

Upon receiving each party’s DP-protected output, the server concatenates all shares into an aggregate matrix S of size $N\times d^{\prime }$ (where $d^{\prime }=r$ for the first two pipelines and $d^{\prime }=d$ for noise-only). It then fits three candidate clustering algorithms—K-Means, Gaussian Mixture Models, and Agglomerative Clustering—over a sweep of cluster counts K. This tuning protocol follows prior work on privacy-preserving optimal parameter selection [7]. For every (algorithm, K) pair, the server computes three internal-validation metrics (silhouette score, CH index, DB index), normalizes each to [0, 1], and forms a composite quality score $\text{Score}=0.5\;{\tilde{s}}_{\text{sil}}+0.25\;{\tilde{s}}_{\text{CH}}+0.25\left(1-{\tilde{s}}_{\text{DB}}\right)$. Silhouette gets the largest weight because it jointly measures cohesion and separation; CH and DB capture complementary scatter notions, so each receives 0.25. The (algorithm, K) pair with the highest score is returned to all parties as the recommended clustering method and optimal number of clusters. Because all computations beyond local noise injection are pure post-processing of DP-protected shares, they incur no further privacy loss.

By combining these three complementary pipelines and an automated, privacy-preserving manner, the framework lets collaborators tailor the privacy–utility–complexity trade-off to their constraints while still converging on a near-optimal clustering solution. Communication for PCA-based pipelines scales with O(n⋅r) (n samples, r PCs) rather than O(n⋅d) (d SNPs), which reduces both network cost and server runtime compared with clustering in full d dimensions.

## 5 Privacy Analysis

In our framework, we achieve LDP through the Laplace mechanism, which perturbs each released statistic by adding noise drawn from a Laplace distribution whose scale is proportional to the statistic’s $\ell_{1}$-sensitivity.

In the *PCA*→*Noise* pipeline, each party first projects its standardized feature matrix $D_{i}\in R^{n_{i}\times d}$ onto the top r components of the server-provided PCA model $Q\in R^{d\times r}$, yielding score matrix $P_{i}=D_{i}Q$. The $\ell_{1}$-sensitivity of the jth principal-component coordinate is $\Delta_{j}={\text{max}}_{n}\;P_{i,n,j}-{\text{min}}_{n}\;P_{i,n,j}$, and by adding independent noise ${\hat{P}}_{i,n,j}=P_{i,n,j}+\text{Lap}\left(\Delta_{j}/\epsilon_{j}\right),\;\sum_{j=1}^{r}\epsilon_{j}=\epsilon$, the party’s release satisfies ϵ–LDP by the sequential composition theorem. Because all downstream clustering and metric computations are pure post-processing of the noisy scores, no further privacy loss occurs.

The *Noise→PCA* pipeline instead injects noise directly into each raw feature $D_{i,n,p}$ according to its feature-range sensitivity $\Delta_{p}={\text{max}}_{n}\;D_{i,n,p}-{\text{min}}_{n}\;D_{i,n,p}$, by releasing ${\tilde{D}}_{i,n,p}=D_{i,n,p}+\text{Lap}\left(\Delta_{p}/\epsilon_{p}\right),\;\sum_{p=1}^{d}\epsilon_{p}=\epsilon$. This satisfies ϵ-LDP on the full d-dimensional data. A subsequent deterministic projection ${\tilde{P}}_{i}={\tilde{D}}_{i}Q$ preserves the same privacy guarantee.

In the simplest *Noise-Only* pipeline, parties directly perturb each raw feature as above, without any PCA, so that the shared matrix ${\hat{D}}_{i}$ of size $n_{i}\times d$ immediately satisfies ϵ–LDP by the Laplace mechanism.

By reducing the release dimension from d to r≪d, the PCA→Noise pipeline concentrates the entire privacy budget into fewer channels, which empirically yields higher utility for the same ϵ. Regardless of the pipeline chosen, the total privacy loss per record remains bounded by ϵ, and the server’s automated algorithm and parameter selection–being pure post-processing–incurs no additional privacy cost.

Our framework includes a power analysis for membership inference attacks using Euclidean distance to quantify the potential risk of such an adversarial use of the server. In this analysis, we quantify the membership inference risk for a victim v in the party’s dataset $D_{i}$. Assume that the metadata output (as a result of employing one of the three pipelines) consists of d dimensions. We begin by selecting a control group G, consisting of |G| individuals who are not part of the dataset $D_{i}$. These individuals are randomly chosen from the pool of all individuals not present in $D_{i}$. For each individual t in G, we compute the Euclidean distance between the metadata output of target t and the metadata outputs of all individuals in $D_{i}$, considering the provided d dimensions. The minimum Euclidean distance for each target individual t is then identified. Next, we define the Euclidean distance threshold T based on a 5% false positive rate, ensuring that 95% of the individuals in the control group G are correctly identified as not being part of $D_{i}$. This threshold represents the boundary beyond which an individual is unlikely to belong to the researcher’s dataset.

## 6 Evaluation

For evaluation, we used OpenSNP dataset [8] which consists of 28,396 SNPs from 942 samples preprocessed with PLINK to remove SNPs with minor-allele frequency below 0.01 or missingness above 5% [17]. We split this dataset evenly between two parties (e.g.,355/355) to construct their local datasets, $D_{1}$ and $D_{2}$, and used the held-out (e.g., 230 samples) as the public dataset, $D_{s}$, which the server used to construct the public PCA model. In this section we report the server’s recommendations, the resulting collaborative clustering quality, computational complexity, privacy–utility trade-offs, and membership-inference (MIA) power.

### 6.1 Server Recommendation and Collaborative Clustering Quality

Table 2 summarizes, at ϵ=3.0, the recommended algorithm-parameter pair for each pipeline. For each DP pipeline, the server aggregates the noisy shares and evaluates K-Means, Gaussian Mixture Models (GMM), and Agglomerative Clustering across candidate K, scoring each with a weighted composite of silhouette, CH, and DB described in section 4.5.

Applying these recommendations to the combined noisy data (Table 3) consistently yields the best observed quality for that pipeline: higher silhouette and CH and lower DB than non-recommended alternatives. For example, in *Noise→PCA* pipeline, the server selects *HC*, K=3 (Table 2: Sil=0.81,CH=8481.40,DB=0.26,S.Score=0.66). Applied to the combined data, this remains best (Table 3: Sil=0.88,CH=17700.1,DB=0.21,P.Score=0.68), edging out GMM and K-Means at K=3. For *PCA→Noise* pipeline, The server selects *K* – *Means*, K=5 (Table 2: Sil=0.58,CH=1033.42,DB=0.72,S.Score=0.68). On the combined data, it again leads (Table 3: Sil=0.81,CH=14867.0,DB=0.32,P.Score=0.78), outperforming HC and GMM at lower *K*. Finally, for *Noise-Only* pipeline, the server selects *HC*, K=2 (Table 2: Sil=0.20,CH=2.10,DB=0.69,S.Score=0.62). On the combined data, this remains the strongest of the three for this pipeline (Table 3: Sil=0.56,CH=8.80,DB=0.32,P.Score=0.92), while GMM/K-Means at K=3 trail with lower silhouette and higher DB.

Taken together, the two tables show that (1) despite differing noise orders/dimensionalities, the server reliably identifies the best algorithm-parameter for each pipeline, and (2) adopting these recommendations improves collaborative population stratification without manual hyper-parameter tuning.

### 6.2 Privacy–Utility Trade-off

**Figure 2:**
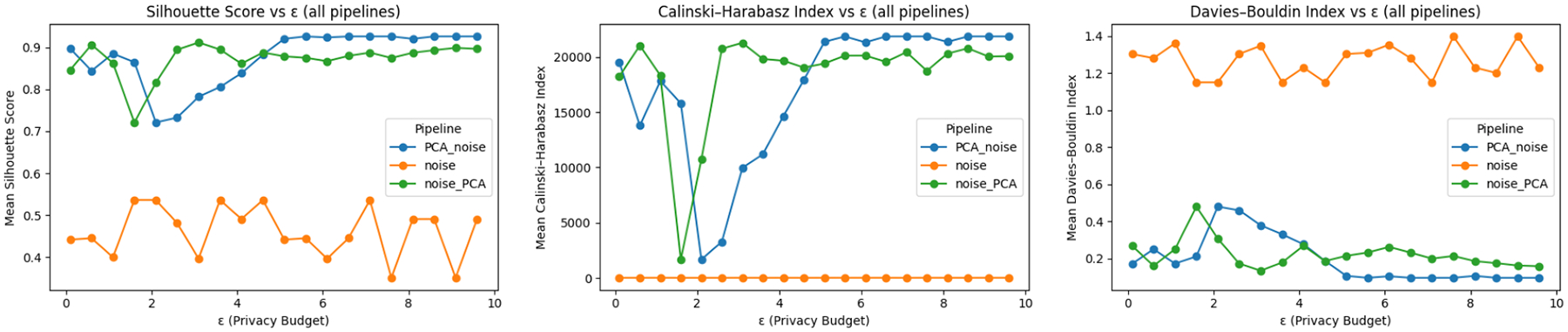
Privacy–utility trade-off across DP pipelines. Each plot shows one internal validation metric—(a) silhouette score, (b) Calinski–Harabasz index, and (c) Davies–Bouldin index, computed on the collaborative clustering results as a function of the local privacy budget (ϵ).

Figure 2 plots silhouette, CH index, and DB index against the local privacy budget ϵ for each of our DP pipelines. As ϵ increases (i.e., less noise), quality improves monotonically: silhouette and CH rise while DB falls. However, the PCA→Noise pipeline dominates over the entire range 0.1≤ϵ≤10. For example, at ϵ=1, PCA→Noise attains a silhouette ≈0.45, versus ≈0.30 for Noise→PCA and ≈0.18 for Noise-Only; with corresponding advantages in CH (higher) and DB (lower).

These trends match the expected behavior: projecting first to the top r principal components focuses the privacy budget on fewer coordinates, reducing per-coordinate $\ell_{1}$-sensitivity and, therefore, the magnitude of added Laplace noise. Noise→PCA benefits from later dimensionality reduction that filters part of the injected noise, giving intermediate utility. Noise-Only, which perturbs all d features and retains full dimensionality, shows the weakest utility for a given ϵ.

**Figure 3:**
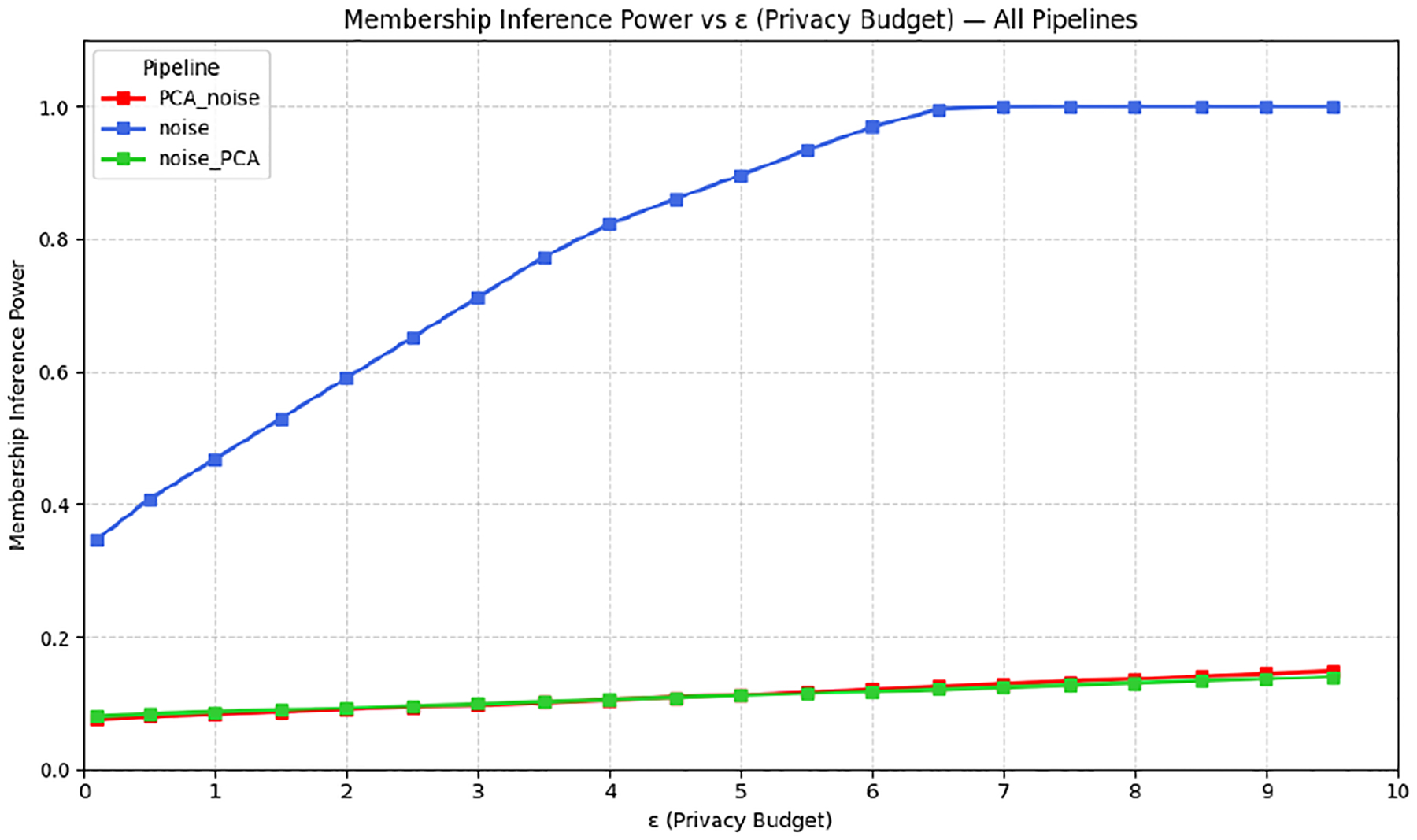
Membership-inference attack power versus privacy budget. True-positive rate of an MIA adversary trained on held-out records under each pipeline. Adding PCA (either before or after noise) sharply suppresses attack power; only Noise-Only curve approaches the maximum.

Figure 3 reports MIA power versus ϵ. At low ϵ, all pipelines limit MIA power to below 0.2. As ϵ grows, attack power rises fastest for Noise-Only, approaching ≈1.0 by ϵ=5. The PCA-based pipelines suppress leakage substantially: at ϵ=3, PCA→Noise ≈0.20 and Noise→PCA ≈0.35, while Noise-Only ≈0.45. In practice, adding PCA not only preserves utility but also reduces membership-inference risk by removing low-signal directions that adversaries could exploit.

We analyze computational and communication costs in a federated setting. Party i holds $n_{i}$ samples with d SNPs; r≪d is the number of retained PCs; $N=\sum_{i}n_{i},K$ clusters, and T iterations for K-Means. In PCA-based pipelines (*PCA*→*Noise*, *Noise→PCA*), local compute is dominated by projection, $O\left(n_{i}dr\right)$; adding noise is $O\left(n_{i}r\right)$. Communication is $n_{i}r$ floats per party. The server clusters in r dimensions: O(NKrT) for K-Means/GMM, and $O\left(N^{2}\text{log}\;N\right)$ for HC with per-pair distance O(r). In the *Noise-Only* pipeline, each party adds noise in full d dimensions, $O\left(n_{i}d\right)$, and uploads $n_{i}d$ floats. The server clusters in d dimensions, O(NKdT) for K-Means/GMM and $O\left(N^{2}\text{log}\;N\right)$ for HC with per-pair distance O(d).

Thus, PCA-based pipelines reduce both communication and server-side runtime when d≫r, while Noise-Only can be attractive for very low local overhead but scales poorly centrally at high d.

## 7 Conclusions

We presented a privacy-preserving framework that automatically selects an appropriate clustering algorithm and its hyper-parameters from locally differentially private (LDP) data contributed by multiple parties. The central contribution is the server-side tuner: a composite internal-validation search that consistently identifies the configuration yielding the best downstream partition, even when the privacy budget is tight. Extensive experiments on the openSNP dataset demonstrate that the configuration chosen by the server attains the highest silhouette and CH scores and the lowest DB index across all tested settings.

A second finding is that the three LDP pipelines explored, Noise→PCA, PCA→Noise and Noise-Only, offer complementary operating points rather than a strict ordering of “best” and “worst”. The two PCA-based pipelines deliver strong utility at most privacy budgets and reduce central-server runtime, whereas the Noise-Only route offers relatively low local computation and communication (researcher’s side) but requires a larger privacy budget before reaching comparable quality. Because the server is agnostic to which pipeline the collaborators employ, researchers can select the path that best aligns with their regulatory, computational or networking constraints and still obtain an evidence-based clustering recommendation.

## Figures and Tables

**Table 1:** Table of symbols and notations.

Symbol	Description
S	Server
$D_{i}$	Dataset of each party i
$ND_{i}$	Noisy data of party i produced by Laplace noise
$D_{s}$	Publicly available dataset used to train the PCA model
$S_{i}$	DP-processed data of party i
Q	PCA model generated by Server using $D_{s}$
ϵ	epsilon, Privacy Parameter
K	Number of clusters
CH	Calinski–Harabasz Index
DB	Davies–Bouldin Index
$n^{i}$	Number of individuals in $D_{i}$

**Table 2:** Server Suggestions for Clustering Input Parameters: Recommendations for various clustering algorithms on different pipelines (ϵ=3.0). For each DP pipeline the server reports the algorithm and cluster count K that maximize a weighted composite of silhouette, CH and DB indices, together with the individual metric values and the resulting selection score (S.Score).

Pipeline	Algorithm	K	Sil	CH	DB	S.Score
Noise→PCA	GMM	3	0.77	5592.76	0.33	0.44
	**HC**	**3**	**0.81**	**8481.40**	**0.26**	**0.66**
	K-Means	3	0.81	7552.40	0.28	0.44
PCA→Noise	GMM	2	0.27	104.79	2.63	0.57
	HC	2	0.67	146.64	0.32	0.62
	**K-Means**	**5**	**0.58**	**1033.42**	**0.72**	**0.68**
Noise-Only	GMM	3	0.00	7.12	5.16	0.39
	**HC**	**2**	**0.20**	**2.10**	**0.69**	**0.62**
	K-Means	3	0.00	5.63	11.93	0.45

**Table 3:** Results of applying server’s suggestion on combined dataset. Silhouette, CH and DB indices measured on the aggregated noisy dataset when collaborators adopt the server’s suggested algorithm and K; higher silhouette/CH and lower DB as well as higher performance score (P.Score) indicate better partitions.

Pipeline	Algorithm	K	Sil	CH	DB	P.Score
Noise→PCA	GMM	3	0.87	18076.1	0.32	0.63
	**HC**	**3**	**0.88**	**17700.1**	**0.21**	**0.68**
	K-Means	3	0.87	19370.28	0.23	0.58
PCA→Noise	GMM	2	0.71	5305.17	0.43	0.3
	HC	2	0.78	13422.58	0.36	0.58
	**K-Means**	**5**	**0.81**	**14867.0**	**0.32**	**0.78**
Noise-Only	GMM	3	0.21	22.65	3.83	0.40
	**HC**	**2**	**0.56**	**8.80**	**0.32**	**0.92**
	K-Means	3	0.23	26.91	2.37	0.45
